# Challenges to research progression among part-time master’s students in nursing in China: a qualitative descriptive study

**DOI:** 10.3389/fpsyg.2026.1868184

**Published:** 2026-10-21

**Authors:** Hong Zhang, Xue Xiong, Liyu Chen, Jun Shen, Shuang Liang

**Affiliations:** 1 Chongqing Nursing Vocational College, Chongqing, China; 2 Department of Nursing, The First Affiliated Hospital of Chongqing Medical University, Chongqing, China

**Keywords:** nursing, part-time master’s students, qualitative descriptive study, reflexive thematic analysis, research barriers, research progression

## Abstract

**Introduction:**

Since 2017, master’s programs in nursing in China have admitted part-time candidates who remain in full-time clinical employment throughout their studies. Slow research progress among these students has become a recognized concern. In this study, slow research progress is conceptualized as a phenomenon arising from the interplay of conditions across several levels. Its observable marker is sustained delay in thesis-related milestones. Drawing on Role Theory and Ecological Systems Theory as sensitizing frameworks, this study describes the challenges that part-time master’s students in nursing encounter in research progression and the multilevel conditions that shape them.

**Methods:**

This qualitative descriptive study was conducted at one medical university in China. Fourteen participants, including two who had withdrawn after exceeding the maximum permitted duration of study, were recruited by maximum-variation purposive and snowball sampling between October and December 2025. Seventeen semi-structured interview sessions, including three supplementary interviews, were analyzed using reflexive thematic analysis. Sample size was judged against the concept of information power. The study is reported in accordance with the Consolidated Criteria for Reporting Qualitative Research (COREQ).

**Results:**

Five themes were developed, namely role conflicts and resource depletion, structural barriers within the microsystem, insufficient support from mesosystem and exosystem levels, macro-level cultural influences, and temporal-developmental constraints. The first four themes were described by participants at every stage of candidature. By contrast, the sequence from interrupted learning through deferral to loss of academic confidence was elaborated mainly by participants in later years and by those who had withdrawn.

**Conclusion:**

Participants’ accounts support treating slow research progress as a trajectory shaped by conditions operating at several levels rather than as an individual trait or behavior. Participants themselves, however, sometimes explained their difficulties in individual terms. The findings indicate that support for part-time postgraduate learners in nursing should not target students alone. It also needs to address the organization of clinical work, the coordination between hospital and university, and the design of the part-time route.

## 1 Introduction

Nursing is a practice-oriented applied discipline. With the rapid global development of the nursing profession, demand for highly qualified, specialized nurses has increased substantially. Cultivating master’s-level nursing students with systematic professional knowledge, advanced clinical reasoning skills, and strong research competence has therefore become critically important (Abu-Qamar et al., 2020). Nursing education in China has expanded from a single secondary technical level to a multi-tiered, diversified system encompassing associate, undergraduate, and master’s degree programs (Shaomei et al., 2021) Master’s education in nursing began in 1992, when Beijing Medical University enrolled its first cohort of master’s students in nursing. Master’s programs have become the primary pathway for training senior nursing professionals in teaching, research, and advanced clinical practice (Song et al., 2024). By 2024, more than 140 universities in China had established master’s programs in nursing (China Graduate Admission Information Network, 2024). This expansion has taken place within a broader growth of postgraduate education. According to the 2024 National Statistical Bulletin on Educational Development, 1.19 million students were newly enrolled in master’s programs in China in 2024. Total master’s enrollment stood at 3.42 million (Ministry of Education of the People’s Republic of China, 2025). Despite this expansion, postgraduate nursing education in China remains at a comparatively early stage of development. The research capacity of postgraduate nursing students, particularly those enrolled part-time, has been reported as relatively limited (Zhi et al., 2018).

Since 2017, master’s programs in nursing in China have admitted part-time candidates, who remain in full-time clinical employment throughout their studies. The number of registered nurses who study in this way has grown accordingly. The part-time program is not a research-based degree. It combines taught courses, concentrated in the first year, with a compulsory research component. The standard duration is 3 years, and the maximum permitted duration is typically 6 years. Graduation requires an independent research project and a thesis that passes formal defense. Part-time students receive no reduction in clinical workload during their studies.

Within this landscape, part-time master’s students in nursing constitute a distinct group. They pursue postgraduate degrees while carrying demanding clinical responsibilities. They are not only a pivotal force in clinical nursing practice but also an important driver of nursing research and disciplinary development. In educational-psychological terms, they are also working adult learners who hold the roles of employee, student, and, in most cases, family member at the same time. Research on working students has shown that demands arising from employment interfere with the student role and predict poorer academic performance and attitudes (Butler, 2007). Studies of registered nurses pursuing advanced degrees have documented work-family-school role conflicts associated with burnout and reduced psychological well-being (Goong et al., 2016; Xu and Song, 2016). Beyond nursing, part-time doctoral students report weaker socialization into the academic community and less access to supervisors and peers than their full-time counterparts (Gardner and Gopaul, 2012)These findings suggest that the difficulties of part-time postgraduate study are not confined to nursing or to China, although the form they take depends on the program and the setting.

Under these conditions, slow research progress among part-time master’s students in nursing has attracted increasing attention in China. Part-time students have been reported to pass thesis proposal reviews and final thesis evaluations at lower rates than full-time students (Wang et al., 2024). Anxiety about delayed graduation also features prominently in their accounts of the research process (Xu et al., 2025). In this study, slow research progress denotes sustained delay in reaching the thesis-related milestones of proposal approval, data collection, thesis submission, and defense within the timeframe expected by the program. Such delay may lead to extended candidature and, where a maximum duration applies, to withdrawal. The term designates neither an individual trait or behavior nor an endpoint. It refers to a trajectory that arises from the interplay of conditions at several levels and unfolds in a particular institutional and social context. Which conditions shape this trajectory, and how, is treated here as an empirical question.

Slow research progress should be distinguished from five related concepts. *Delayed research completion*, typically indexed by time-to-degree (de Valero, 2001) describes only the endpoint of lateness. It says nothing about the trajectory that produced it. *Academic procrastination* refers to the voluntary postponement of an intended action even though the person expects the delay to leave them worse off (Steel, 2007) It locates the problem within the person. *Low research engagement* is a psychological state that can form part of, or feed into, slow research progress. Engagement denotes the behavioral, emotional, and cognitive investment a student brings to academic work (Kahu, 2013). *Reduced research self-efficacy* can play a similar part. *Research self-efficacy* denotes confidence in one’s ability to carry out specific research tasks (Bieschke et al., 1996). Both states are narrower than slow research progress itself. Finally, *slow scholarship* is a movement in Western academia that advocates deliberately unhurried, reflective inquiry as a counter to productivity pressures (Hartman and Darab, 2012). Because it is an intentional stance, it differs in kind from the involuntary delay examined here.

A scoping review of the international literature identified six groups of factors that influence postgraduate nursing students’ study progress. These were supervisory and peer support, work-life balance, funding, research writing, program structure, and geographical factors (Mudzi and Sepeng, 2023). Most primary studies in that review drew on mixed samples of full-time and part-time students. Part-time status itself appeared among the factors associated with slower progress. Qualitative work in South Africa has similarly described postgraduate nursing students’ difficulty in balancing employment, family responsibilities, and study, alongside limited supervisory time and delayed feedback (Havenga and Sengane, 2018). In China, a configurational analysis of part-time master’s students in nursing identified research motivation, faculty-student interaction, a supportive work environment, and time management as conditions for learning engagement (Wang et al., 2024).

Taken together, these studies leave three gaps. First, part-time students have seldom been studied as a group in their own right. In most studies they form a subgroup within mixed samples. The consequences of holding a full clinical post throughout candidature are therefore not examined directly. Second, the evidence from China is sparse. The single most relevant study excluded students who had not obtained the degree within the permitted period (Xu et al., 2025). The experiences of students whose delay ended in withdrawal therefore remain undocumented. Third, existing studies have largely produced lists of contributing factors. Less attention has been paid to how these factors are experienced in relation to one another, and to how they bear on the student as a learner. The present study addresses these gaps in three ways. It focuses exclusively on part-time master’s students in nursing. It includes students who had withdrawn after exceeding the maximum permitted duration. It also approaches their accounts with frameworks that direct attention to roles and to context. Its focus on the student as a learner also links the study to educational-psychological work on working adult learners, role conflict, and academic self-efficacy.

This study drew on Role Theory (Biddle, 1986) and Ecological Systems Theory (Bronfenbrenner, 1979) as sensitizing frameworks. Both frameworks were specified *a priori*. They guided what the analysis attended to but did not supply a coding frame. Role Theory directed attention to conflicts among the roles of clinical nurse, research learner, and family member. Studies of working students and of nurses pursuing advanced degrees identify these conflicts as central to part-time study (Butler, 2007; Goong et al., 2016; Xu and Song, 2016). Ecological Systems Theory directed attention to the micro-, meso-, exo-, macro-, and chronosystem levels at which the relevant conditions may operate. This perspective suits students whose study is distributed across a hospital and a university that are separately governed.

Accordingly, this qualitative descriptive study aimed to describe the challenges that part time master’s students in nursing in China experience in making research progress, and the conditions in which these challenges arise. The findings are intended to inform the design of supervisory and institutional support for part-time postgraduate learners in nursing. They may also be relevant to other practice-based disciplines, and to settings outside China where students remain in full-time clinical employment while they study.

## 2 Methods

### 2.1 Study design

The aim of this study was to describe the challenges that part-time master’s students in nursing encounter when attempting to progress with their research, together with the conditions in which those challenges arise. A qualitative descriptive design was adopted for this purpose. Qualitative description is suited to research questions that call for a comprehensive, low-inference summary of an experience, presented in language that stays close to the words and meanings of participants (Sandelowski, 2000). It is widely used in nursing and health research where the intended product is a straightforward account of who experiences what, in which circumstances (Bradshaw et al., 2017; Kim et al., 2017). This design also suits a sample whose members stood in different positions relative to the phenomenon. The present sample included enrolled students at several stages of candidature and students who had withdrawn. Reporting follows the COREQ checklist (Tong et al., 2007).

### 2.2 Recruitment and sampling

Participants were recruited from October to December 2025 using maximum-variation purposive sampling (Palinkas et al., 2015). Snowball sampling (Abrams, 2010) was added to reach students who had left their program and were no longer in regular contact with the university. The aim was to describe the range of challenges that arise across the trajectory of part-time postgraduate study. Variation was therefore deliberately sought in stage of research progression, as well as in gender, age, years of clinical experience, professional position, and clinical department. Participants were recruited from every stage of the program, from the early years of candidature to withdrawal after the maximum permitted duration had been exceeded. Year of study refers to the number of academic years the participant had completed at the time of the first interview. Sampling students at different points of progression, instead of restricting recruitment to those whose candidature had already lapsed, was a deliberate design choice. It allowed difficulties that emerge early in the program to be described alongside those reported by students whose progress had stalled or ended. It also made it possible to examine how comparable challenges were expressed at different stages.

The inclusion criteria were: (1) currently enrolled part-time master’s students in nursing, or former students who had withdrawn after exceeding the maximum permitted duration of study; (2) at least 3 years of clinical nursing experience; (3) completion of at least one academic year of postgraduate coursework; and (4) self-reported experience of slow research progress. To establish self-reported slow research progress, enrolled candidates were asked whether they were behind the milestone schedule set out in their training program. Former students were asked whether their withdrawal had followed a failure to complete the required milestones within the permitted period. The exclusion criteria were: (1) students on a leave of absence, because they were not undertaking coursework or research during the recruitment period; and (2) those with severe language impairment or communication difficulties.

Sample size was judged against the concept of information power (Malterud et al., 2016). This concept was preferred to data saturation, which fits poorly with reflexive thematic analysis (Braun and Clarke, 2006). In reflexive thematic analysis, themes are generated through the researchers’ interpretation rather than discovered in the data. There is therefore no fixed point at which new data stop yielding new meaning. Information power instead judges sample size by how much information relevant to the aim the sample holds (Malterud et al., 2016). In this study, the focused aim, a (Malterud et al., 2016) t experience of slow research progress, and the use of established sensitizing frameworks each increased information power. Recruitment continued until the dataset included participants at each stage of the program and the team judged the material sufficient to address the aim. Recruitment closed after 14 participants had been interviewed. With three supplementary interviews, the dataset comprised 17 interview sessions.

### 2.3 Data collection

Data were collected through semi-structured interviews, conducted face to face except for two supplementary interviews held by telephone. Guided by the study aim, we reviewed relevant literature (Shaomei et al., 2021; Xu et al., 2025) and the training program for part-time master’s students (Graduate School of Chongqing Medical University, 2025). We then consulted two nurse educators with experience of supervising part-time master’s students and one qualitative methodologist to develop a preliminary interview guide. Three part-time master’s students in nursing were then selected for pilot interviews. The guide was revised based on their feedback. Pilot interview data were not included in the analysis. The final guide is shown in Table 1. Interviews were conducted in Mandarin Chinese. The extracts reported in this article were translated into English by a bilingual member of the research team and checked against the original transcripts by a second bilingual member.

**Table 1:** Interview guide for part-time master’s students in nursing.

No.	Interview question
1	What motivated you to pursue a master’s degree in nursing while continuing to work?
2	At the beginning of your program, what plans did you make for your research tasks and timeline? In what ways does your actual research progress differ from your initial expectations?
3	How do you allocate your time and energy among clinical duties, coursework, research activities, and personal life? When conflicts arise among these commitments, how do you usually make decisions or trade-offs? Where does research rank among your overall priorities?
4	How do you define or understand slow research progress? Based on your own experience, can you describe a specific situation in which your research progressed slowly? What direct or indirect factors contributed to this?
5	When experiencing slow research progress, what coping strategies have you used? How effective have these strategies been in addressing the challenges you faced?

Before the interviews, the research team contacted participants via WeChat. The study’s purpose, procedures, confidentiality measures, and participants’ rights were explained in detail. Interview times were then scheduled. Voluntary participation was emphasized. Participants were clearly informed that they could discontinue or withdraw from the interview at any time. All first interviews were conducted face to face in a quiet, private conference room at the participant’s workplace. Participants received no financial compensation.

Two researchers with systematic training in qualitative research methods jointly conducted the interviews. The lead interviewer (HZ; female, Master of Nursing) had extensive experience in both clinical nursing and qualitative research. She asked the questions and conducted in-depth probing. The assistant researcher (SL; female, Master of Education) had substantial expertise in educational studies. She observed and recorded participants’ nonverbal behaviors, including facial expressions, gestures, and variations in intonation. Both interviewers were aware that they shared a professional background with participants. They used this awareness to notice and follow up statements that might otherwise have been taken as self-evident. Open-ended questions encouraged participants to express themselves fully. Techniques such as clarification, summarization, and purposeful pauses were used to deepen understanding. The nonverbal observations served as supplementary data for interpreting participants’ accounts.

Each participant gave written informed consent before the first interview. All interviews were audio-recorded and transcribed verbatim within 24 h of completion. The transcripts included timestamps, notes on emotional changes, and key nonverbal behaviors. In total, 17 interview sessions were recorded. Fourteen were first interviews, one with each participant, conducted between October and December 2025. They ranged from 27 to 39 min (mean 33 min).

The first interviews were relatively short because they addressed a single, well-defined phenomenon. Participants had already reflected on it at length, and their accounts were specific and detailed. Interviews were conducted in parallel with the preliminary data analysis. Questions raised by earlier interviews were pursued in later ones through additional probes on the timing of milestones and on the sources of support participants had sought. Where the preliminary analysis identified gaps, these were addressed by supplementary interviews rather than by extending the first interviews.

The three supplementary interviews were each held with a different participant. They took place after the first round of interviews had been completed and before member checking was concluded in February 2026. They were arranged because the preliminary analysis of the first interviews had raised questions that the existing transcripts could not answer. For two participants, these questions concerned the sequence and timing of the milestone delays that preceded the end of candidature. For one participant, they concerned the sequence of an early interruption and later methods training. One supplementary interview was conducted face to face at the participant’s workplace. The other two were held by telephone, at the participants’ request. Each lasted between 12 and 20 min and followed a short guide written for the individual participant. Each was audio-recorded and transcribed verbatim within 24 h. The transcript was then appended to that participant’s first transcript, so that the two were coded together as a single case record. Codes and candidate themes affected by the additional material were revised accordingly. The three participants concerned were among those invited to member checking.

### 2.4 Data analysis

Data were analyzed using reflexive thematic analysis (Braun and Clarke, 2006). Three members of the research team undertook the analysis. HZ, the lead interviewer, is a registered nurse with a master’s degree in nursing and extensive experience in clinical nursing and qualitative research. SL, the assistant researcher, holds a master’s degree in education. JS, dean of the nursing vocational college, is an expert in geriatric nursing and nursing education. Coding was carried out manually. Annotated transcripts and a shared spreadsheet were used to record codes and their locations. No qualitative data analysis software was used. Analysis was recursive, and the team moved back and forth between phases as the account developed.

Phase 1: Familiarization with the data. The three analysts read the complete dataset repeatedly, together with the field notes on nonverbal behavior and their own reflexive journal entries. They recorded initial analytical impressions and questions rather than formal codes.

Phase 2: Coding. The three analysts independently coded the entire dataset line by line, generating both semantic codes (close to participants’ explicit statements) and latent codes (capturing implicit assumptions and framings). Reflexive thematic analysis does not use a separate meaning-unit stage, so codes were attached directly to segments of transcript text. Coding was open and iterative. Codes were added, merged, split, and relabeled as work proceeded across the corpus. Every transcript was coded by all three analysts.

Phase 3: Generating initial themes. The analysts met to compare and pool their coding. Consistent with the assumptions of reflexive thematic analysis, these meetings were not used to establish coding reliability or to eliminate divergence in favor of a single correct code. Differences between analysts were treated as an analytical resource. Points of disagreement were examined to surface the assumptions underlying each reading. Codes sharing a central organizing concept were then collated into clusters, from which candidate themes and sub-themes were constructed. The thematic structure was settled through discussion and adopted once all three analysts accepted it.

Phase 4: Developing and reviewing themes. Candidate themes were checked first against their collated coded extracts and then against the full dataset. The aim was to confirm that each theme was supported across cases and that the thematic structure as a whole told a coherent story about the data. At this stage the team also examined how the themes related to one another. A cross-stage matrix was constructed in which candidate themes formed the rows and participants the columns, with participants grouped by stage of research progression (years 1–2; years 3–5; withdrawn). The matrix was used to examine patterning across the sample, not to count occurrences. A theme was retained on the basis of its coherence and its relevance to the research question, not the number of participants who contributed to it. Reading across the matrix showed which candidate themes were expressed throughout the sample and which were elaborated mainly at particular stages. It also allowed the team to consider how a challenge described early in candidature related to accounts given by participants further on. Relationships between themes were sought in two ways. First, the team looked for passages in which participants themselves linked one challenge to another. Second, it read the matrix across stage bands for patterns that participants had not stated directly. A relationship was retained only if it could be traced to such a passage or pattern. Retained relationships were represented in a thematic map, which was redrawn several times in team discussion as the boundaries between themes were revised.

Phase 5: Refining, defining, and naming themes. Each theme was defined in a short written statement specifying its scope, its boundary with adjacent themes, and the aspect of the research question it addressed. Each was given a name conveying its content. Integration and interpretation were achieved at this stage through team discussion, in which the defined themes were mapped onto the sensitizing frameworks. Each theme was located at the level of the ecological system to which it principally referred (microsystem; mesosystem and exosystem; macrosystem; chronosystem). Each was also related to the role-theory constructs of role overload, role conflict, and role transition. Two experienced qualitative researchers from outside the project then reviewed the theme definitions, the thematic map, and the supporting extracts. They acted as critical friends. Their role was to probe the coherence and plausibility of the analysis and to require the team to articulate the reasoning behind its interpretive choices. It was not to verify the accuracy of the themes. Theme definitions were refined in response. Table 2 illustrates this pathway for one extract from each theme.

**Table 2:** Illustrative pathway from interview extract to code, sub-theme, and theme (one example per theme).

Extract (abridged)	Initial codes (condensed)	Sub-theme	Theme
“My research time is fragmented into scattered moments, making it nearly impossible to secure a solid block of time to focus on academic work.” (P3)	Research time fragmented; no protected block for study	1.1 Role Overload and Fragmented Time Allocation	1 Role Conflicts and Resource Depletion
“Our department is seriously understaffed. … there’s no energy left for research or identifying new problems.” (P9)	Understaffing; no energy left for research	2.1 Systemic Constraints in Clinical Practice Settings	2 Structural Barriers within the Microsystem
“I worked consecutive shifts … I had to rush straight to class.” (P7)	Shift rota not coordinated with class timetable	3.1 Absence of Cross-Institutional Coordination Mechanisms	3 Insufficient Support from Mesosystem and Exosystem Levels
“… the head nurse basically made it mandatory for me to enroll. It wasn’t something I chose.” (P5)	Enrollment required by employer; little personal interest in research	4.2 Utilitarian Academic Culture and Externally Driven Participation	4 Macro-Level Cultural Influences
“After so much fragmented and interrupted learning, I started putting things off more and more. The longer I delayed, the less confident I felt.” (P13)	Interrupted learning; postponing tasks; falling confidence	5.3 Interrupted Learning, Eroding Academic Confidence, and Deferral of Research	5 Temporal-Developmental Constraints

Phase 6: Writing up. Extracts were selected to illustrate each theme and embedded in an analytical narrative. The themes were then related to the wider literature. Writing was treated as part of the analytical process. The thematic structure continued to be refined during drafting.

### 2.5 Rigor and trustworthiness

Rigor was addressed with reference to the criteria of credibility, dependability, confirmability, and transferability (Lincoln and Guba, 1983) and through reflexivity.

Reflexivity and researcher positionality. The five-member research team was drawn from a nursing vocational college and from the department of nursing of a Grade A tertiary teaching hospital. Three members are nurse educators, and two hold clinical or nursing management posts. One of the nurse educators also holds a master’s degree in education. All five members had previously conducted qualitative studies. The team therefore shared with participants a professional world of shift work, clinical performance targets, and hospital hierarchy. Several members were known to participants as colleagues. This insider position (Berger, 2013) cilitated access and candor. It also carried two risks. Assumptions held in common, for example about what constitutes a reasonable clinical workload or what supervisors ought to provide, might pass unexamined. Participants might also frame their difficulties in terms they expected the team to endorse. The team sought to make these preconceptions visible and to work with them. Each researcher recorded expectations, reactions, and points of surprise in a reflexive journal before and after each interview and throughout coding. Journal entries were brought to analysis meetings and used to question readings that had seemed self-evident to the team. Interviews were conducted by researchers who held no supervisory, evaluative, or line management relationship with any participant. This was stated explicitly to participants before consent was obtained.

Credibility. Interview recordings, field notes, and reflexive journals were analyzed together. This allowed the accounts to be cross-checked. Preliminary themes were returned to four participants for feedback. They were chosen to include at least one participant from each stage band, among them one who had withdrawn. Member checking was used to enrich and sharpen the account rather than to validate it (Birt et al., 2016). In response, the wording of three theme labels was refined. Contact with participants and the data extended over a period of about 5 months, from the first interview in October 2025 to the conclusion of member checking in February 2026. The researchers’ long-standing presence in the professional setting also provided the familiarity with context that prolonged engagement is intended to secure. The analysis was opened to challenge by the two external qualitative researchers, who reviewed the design, the thematic structure, and the use of the theoretical frameworks.

Dependability. An audit trail was maintained, comprising the audio recordings, verbatim transcripts, successive versions of the coding framework and thematic maps, minutes of analysis meetings, and the reflexive journals. It allows an independent reader to follow the pathway from raw data to reported themes.

Confirmability. The reflexive journals and the audit trail together allow the interpretations presented here to be traced back to the interview data and to the analytical decisions from which they arose. Illustrative extracts are reported for every theme, allowing readers to assess the fit between data and interpretation.

Transferability. The setting, the structure and requirements of the training program, and the institutional context are described in detail. Readers can therefore judge how far the findings may inform other settings. Participant characteristics are reported in aggregated form. This limits contextual detail but protects confidentiality in a small and identifiable population.

### 2.6 Ethics

This study followed the latest revision of the Declaration of Helsinki issued by the World Medical Association. The study protocol was approved in 2023 by the Ethics Committee of the First Affiliated Hospital of Chongqing Medical University (approval No. 2023–035). Data were collected from October 2025 to February 2026 in accordance with the approved protocol, with no change to the study population, aims, or interview procedures. All participants voluntarily signed a written informed consent form before their first interview, after being fully informed of the study’s purpose, procedures, and potential risks and benefits.

Several safeguards protected participant privacy. Interview recordings and transcripts were pseudonymized by replacing real names with participant codes (P1-P14), assigned in random order. Only the lead researcher held the key linking codes to identities. In the extracts reported in this article, identifying details such as children’s ages, clinical specialty, department, recent promotions, and the length of an absence were removed or generalized. Electronic data were stored in encrypted form. Hard-copy documents were kept in a locked cabinet accessible only to core research team members. Participants were told that they could withdraw at any stage without negative consequences.

## 3 Results

### 3.1 Participant characteristics

Fourteen part-time master’s students in nursing took part, 12 women and 2 men. Seven were aged 31–35 years, and seven were aged 36 years or older. All had more than 10 years of clinical experience, and half held a head-nurse or equivalent nursing management post. Eleven had at least one child under 18 years of age. At the time of the first interview, six participants were in their first or second year of candidature and six were in their third to fifth year. Two had withdrawn from the program after exceeding the maximum permitted duration of study. Table 3 reports participant characteristics as aggregated category frequencies.

**Table 3:** Characteristics of participants (*n* = 14).

Characteristic	Category	*n*
Gender	Female	12
	Male	2
Age group (years)	31–35	7
	≥36	7
Marital status	Married	11
	Not currently married	3
Dependent children	With minor children	11
	No minor children	3
Nature of clinical role	General clinical care	5
	Specialized care	2
	Managerial	7
Clinical experience (years)	>10	14
Stage in master’s program	Years 1–2	6
	Years 3–5	6
Withdrawn (2)		

### 3.2 Themes

Five themes, comprising 14 sub-themes, were developed from the interviews. Theme names carry the ecological level to which each theme was mapped at the theme-naming stage of the analysis. The level labels were added after the themes had been generated and were not used in coding. In this study, the microsystem refers to the settings in which participants took part directly, namely the ward, the supervisory relationship, and the peer group. The mesosystem refers to the links, or their absence, between the hospital and the university. The exosystem refers to arrangements that affected participants without their involvement, such as curriculum design and institutional procurement of resources. The macrosystem refers to prevailing beliefs about credentials and about the standing of nursing research. The chronosystem refers to the timing and sequence of events over the course of study. The five themes are Role Conflicts and Resource Depletion, Structural Barriers within the Microsystem, Insufficient Support from Mesosystem and Exosystem Levels, Macro-Level Cultural Influences, and Temporal-Developmental Constraints. Figure 1 shows their placement within the ecological systems framework and the relationships among them that participants described or that were read from the cross stage matrix.

**Figure 1:**
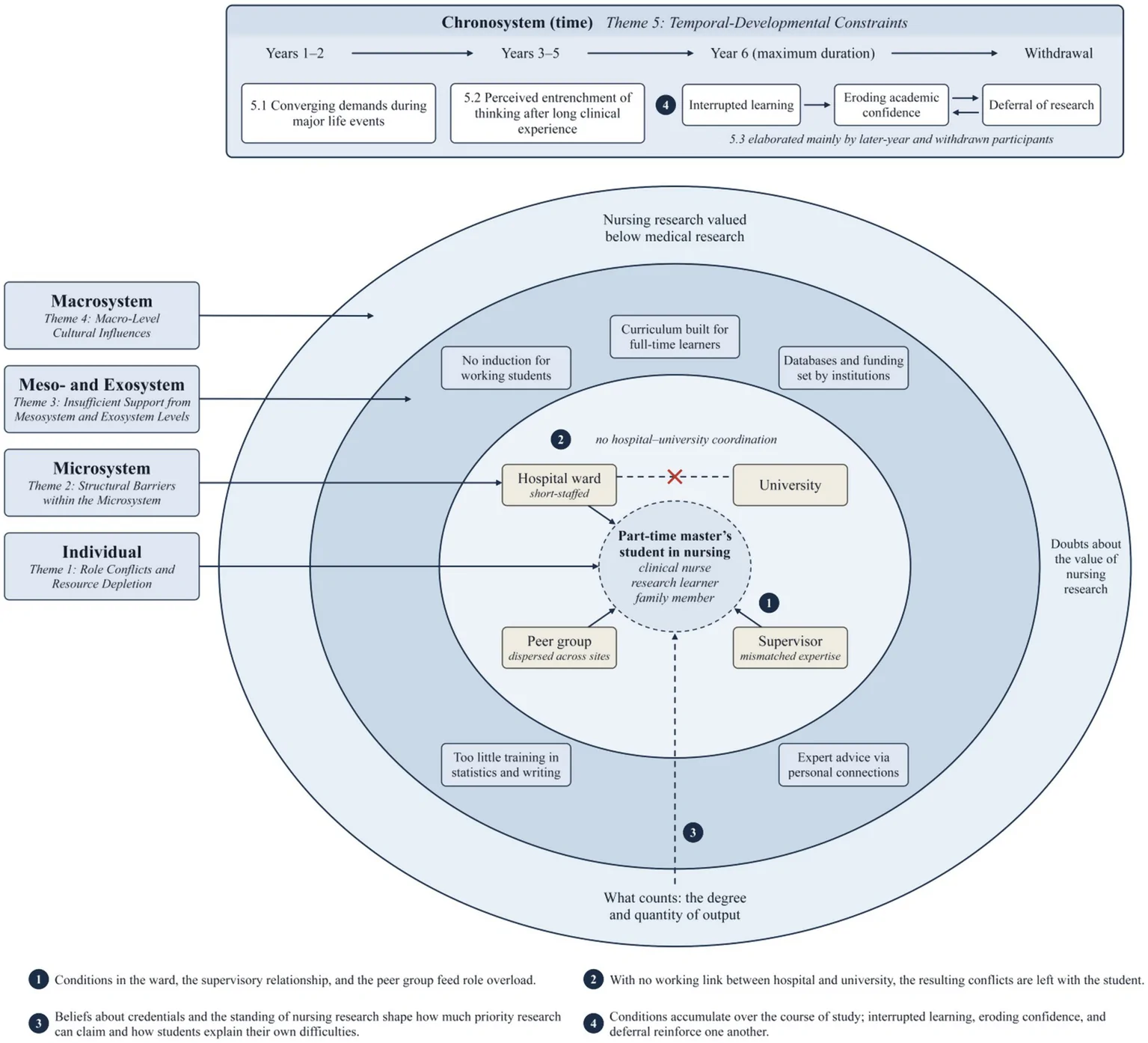
Conceptual map of the five themes within the ecological systems framework.

#### 3.2.1 Theme 1: role conflicts and resource depletion

##### 3.2.1.1 Sub-theme: role overload and fragmented time allocation

Participants described holding several roles at once, including those of clinical nurse, graduate student, and family caregiver. They said they were expected to meet all three sets of demands at the same time. They described their time and energy as insufficient for these demands taken together. Trying to meet professional, academic, and family obligations, they described considerable pressure and persistent difficulty in keeping the three roles in any workable balance.

“*Our clinical workload is overwhelming. It is common to continue emergency care from the day shift late into the evening, sometimes until eight or nine o’clock. On one hand, I am responsible for departmental quality control and clinical precepting; on the other, I must also supervise my child’s studies at home. It truly feels as though I am being pulled apart into several pieces.” (P5).*

Participants also described electronic medical record systems and mobile health tools as streamlining some workflows. They said these tools also added continuous documentation and messaging demands that further fragmented their time.

*“Nowadays, various electronic systems have made clinical procedures increasingly granular. Simply managing quality control data and preparing meeting minutes consumes a significant portion of my day. On top of that, I must constantly monitor my phone to respond to messages. My research time is fragmented into scattered moments, making it nearly impossible to secure a solid block of time to focus on academic work” (P3).*

##### 3.2.1.2 Sub-theme: frequent role switching and mental exhaustion

Participants described frequent interruptions and shifts among clinical, academic, and family tasks as leaving them mentally depleted and unable to concentrate on research.

*“Sometimes, just as I come up with an idea and begin searching the literature, I get interrupted by something else and have to take care of that instead. These interruptions occur frequently. I often feel as though my train of thought has been fragmented, making it really difficult to concentrate on research” (P12).*

Participants also described irregular work schedules, and night shifts in particular, as producing sleep loss and lasting physical fatigue. They attributed impaired attention, memory lapses, and reduced performance to this fatigue.

*“Working night shifts for an extended period really takes a toll. I feel that my memory and ability to learn have both deteriorated. Sometimes, I walk to a place and suddenly forget why I went there. This has already had a serious impact on my daily work and life” (P6).*

##### 3.2.1.3 Sub-theme: conflicting role expectations and contradictory value orientations

Participants described clinical nursing practice and academic research as pulling them toward divergent value orientations. In their experience, clinical work rewarded immediacy and the efficient resolution of practical problems. Academic research, by contrast, demanded theoretical reflection and the systematic exploration of underlying patterns. They described themselves as caught between clinical duties and academic research. They spoke of conflicting commitments and of a strain that accumulated over time.

Participants located this conflict most sharply in their exchanges with supervisors.

*“We initially hoped that graduate study would help us solve practical problems in our daily work, but our supervisors kept emphasizing the need to explore the underlying scientific principles. It feels like being someone who puts out fires in the ward every day and is suddenly asked to stop everything to investigate the causes of the fire. In the end, you are stuck, unable to move forward in either direction” (P10).*

Participants described continually negotiating between these expectations and deciding how to allocate limited time and energy. When clinical demands dominated, research was the commitment they set aside.

*“As part-time master’s students in nursing, we have to juggle work and family responsibilities. Once the clinical pace intensifies, there’s virtually no time left for research” (P1).*

#### 3.2.2 Theme 2: structural barriers within the microsystem

##### 3.2.2.1 Sub-theme: systemic constraints in clinical practice settings

Participants described their departments as chronically short-staffed. They said that on both day and night shifts, each nurse covered more patients than they could manage while also protecting time for study.

*“Our department is seriously understaffed. During the day, one nurse has to manage up to 10 beds, and the night shift is even more overwhelming. Just finishing the daily tasks feels like an achievement, and there’s no energy left for research or identifying new problems” (P9).*

Participants described the hospital environment as consuming not only their time but also the mental resources they needed for research.

*“In my department, we are on duty from morning until late in the evening, overwhelmed by numerous and fragmented clinical tasks. After an entire day of nonstop work, I feel utterly exhausted and unable to concentrate on any research” (P14).*

##### 3.2.2.2 Sub-theme: ineffective supervisor–student interactions

Participants regarded supervision as central to their progress. They described three recurring problems, namely prolonged topic negotiation, a mismatch between supervisors’ expertise and their clinical research interests, and guidance that did not connect clinical experience with research. One participant’s account of a year-long exchange with her supervisor contained all three.

*“I spent an entire year struggling with my supervisor just to get a topic approved. She insisted I work on her specialty, but I neither understood it nor had the capacity to pursue it. What I really needed was someone to guide me in transforming clinical experience into research, but that kind of support was completely lacking. The more I tried, the more confused I became” (P8).*

Participants also described the mismatch between a supervisor’s field and their own clinical research interest on its own. They said it limited how far the supervisor’s guidance could be applied.

*“My research focuses on a clinical nursing specialty, but my supervisor specializes in basic science. She tries to offer guidance, but she does not understand many aspects of clinical practice” (P10).*

##### 3.2.2.3 Sub-theme: weakening of peer support networks

Participants said that when problems arose in their research, they had no fellow students to turn to. They were left to work things out on their own. Because they were dispersed across different posts and institutions, participants said they had no formal channel through which to communicate or work together. They described few opportunities for timely exchange of knowledge or for shared momentum.

*“We part-time master’s students in nursing are all based in different institutions, and we are each tied up with our own clinical duties. Unlike full-time students, we don’t have the kind of environment where we can work on our project together every day. When we run into problems, we don’t even know who to turn to. Most of the time, we are left fumbling our way through on our own” (P11).*

#### 3.2.3 Theme 3: insufficient support from mesosystem and exosystem levels

##### 3.2.3.1 Sub-theme: absence of cross-institutional coordination mechanisms

Participants described hospitals, departments, and universities as operating largely independently of one another. When schedules and resources were not coordinated across these sites, participants reported commuting repeatedly between them. They described this as physically and mentally draining. They also said the arrangement offered little structured academic support.

*“During my first year of graduate school, I worked consecutive shifts, starting with an 8 a.m. to 4 p.m. day shift followed immediately by a night shift. After that, I didn’t even dare to rest. I had to rush straight to class. Trying to study anything systematically under those conditions was nearly impossible.” (P7).*

##### 3.2.3.2 Sub-theme: curriculum and support structures designed for full-time learners

Participants described two distinct problems with the way the program was organized for students who were also working. The first concerned what was taught. Participants said the course content stayed at a general level, was rarely connected to their clinical work, and gave them too little methodological training to carry out a study of their own. The second concerned what was not provided at all. Participants said there was no systematic induction for students entering the program from full-time employment. They had to work out the first steps of a research project alone.

On course content, participants said the curriculum gave them too little training in statistics, academic writing, and critical appraisal. What they had been taught did not carry over to what their own projects required.

*“The curriculum design at school makes little sense. Most of the content is very superficial, and I didn’t actually learn much. For example, when it comes to statistics, I had no idea how to apply it when I actually needed it” (P2).*

In the absence of any formal induction, participants described assembling what they needed by themselves or from classmates.

*“Honestly, I think the biggest issue is that no one guides us from the beginning. As parttime students, we have to piece things together on our own. Whether it’s searching for literature or working on a research topic, we do everything by ourselves. Even getting a bit of advice from a classmate feels like rare help” (P1).*

##### 3.2.3.3 Sub-theme: inequitable access to research resources

Participants described their access to research resources, in particular literature databases and research funding, as determined by their hospital and university. They said they had no influence over these decisions. Restricted access to information systems was a common complaint. Participants also described interruptions in subscription renewals as complicating their efforts to keep their nursing knowledge current and to sustain their research.

*“Our hospital used the Wanfang database for several years, but it required an annual renewal. Later, the hospital stopped paying for it, so now the content is outdated, and we often can’t even connect to it” (P13).*

Participants also described access to good materials and to expert advice at the proposal and thesis-writing stages as depending on personal connections. They said there was no formal provision to fall back on.

*“You can ask around, how many nursing supervisors are really willing to share their grant proposals for you to learn from? Medical supervisors might be open to sharing, but in nursing, if you don’t have the right connections, no one will give you anything” (P1).*

#### 3.2.4 Theme 4: macro-level cultural influences

##### 3.2.4.1 Sub-theme: disciplinary identity crisis regarding the value of nursing research

Participants said that physicians, and their hospitals more broadly, regarded nursing research as less significant than medical research. They spoke of a diminished sense of professional identity and worth. They also described their work as rarely translated into clinical change or recognized beyond nursing. In their accounts, this fragile sense of disciplinary identity went together with reduced motivation to take on research. It also went together with growing uncertainty about whether nursing scholarship was worth doing.

*“As clinical nurses doing research, we are often told that we’re just solving “work problems” instead of addressing real “scientific problems.” Over time, even we begin to question whether nursing research really matters. You look at the doctors, their research topics have clear significance. In contrast, our work feels like we’re just talking to ourselves, and in the end, our results don’t even connect back to clinical practice” (P1).*

##### 3.2.4.2 Sub-theme: utilitarian academic culture and externally driven participation

Participants described an evaluation system in which what counted was obtaining the degree and producing a countable quantity of research output. They described their engagement with research as driven by external requirements and seldom by interest in the work itself. These requirements included degree completion, professional title advancement, performance review, and the expectations of supervisors and employers.

*“The whole environment is incredibly competitive right now, and our supervisors keep pushing us. On top of that, educational requirements keep rising. I went to graduate school just to keep up with the trend” (P10).*

Participants who described their studies in these terms also said they had little to draw on once the work became difficult. They described letting it go instead of pressing on.

*“The problems I’ve had with research probably come down to me. I’ve never been that interested in it to begin with, and the head nurse basically made it mandatory for me to enroll. It wasn’t something I chose. At this point, I just hope I can graduate. And if I can’t, then so be it” (P5).*

#### 3.2.5 Theme 5: temporal-developmental constraints

##### 3.2.5.1 Sub-theme: converging demands during major life events

Participants who were navigating major life events, such as pregnancy, childbirth, or promotion, described these periods as times when academic, family, and professional demands converged. Participants who were pregnant or caring for infants described the sharpest version of this convergence. They described competing demands on time and energy that made sustained participation in coursework and research impossible for a period. Taught courses are concentrated in the first year. When such a period coincided with that year, participants described missing foundational training that was not subsequently made up.

*“When I was pregnant and recovering after childbirth, I missed several weeks of classes at the start of my first year. I never received systematic training in basic research methods like literature searching. What I use now is just a set of Chinese search strategies I figured out myself, which is completely different from what is formally taught” (P1).*

One participant described carrying the primary responsibility for caregiving and domestic work alongside clinical and academic demands. She had also recently taken on new managerial responsibilities.

*“I have two young kids. The younger one is still an infant. I recently took on a new management role and have been trying to learn the ropes in the new role. By the time I get home in the evening, I can barely find a quiet moment to read the academic literature. Sometimes I feel like being in graduate school is more like fighting a guerrilla war” (P1).*

##### 3.2.5.2 Sub-theme: perceived entrenchment of thinking after long clinical experience

Participants described their long clinical experience as working in two directions. It gave them a supply of practical problems and a sense of what mattered in practice. In their accounts, it also entrenched the way they approached problems and made new lines of thinking harder to open. Participants described clinical settings as placing a strong emphasis on standardized procedures and operational protocols. They regarded these norms as essential for ensuring patient safety. They also described them as contributing to rigid adherence to established methods.

*“As clinical nurses, we become increasingly mechanical over time, just sticking to the way things were always done. After a while, our thinking gets boxed in, and it becomes very difficult to come up with new ideas” (P7).*

Participants described the overarching responsibility for patient safety in daily clinical practice as compelling them to prioritize error-free performance and minimize risk. Participants associated this emphasis on zero tolerance for mistakes with strict adherence to standardized procedures. In their own accounts, they also associated it with a habitual preference for stability and caution. They described innovative exploration as feeling incompatible with the realities of clinical responsibility.

*“Once you’ve been in the clinic long enough, your thinking starts to solidify. You begin to believe that “this is just the way things are done,” and you don’t dare to try anything new. After all, we’re dealing with human lives, who would take that kind of risk with patient safety?” (P9).*

##### 3.2.5.3 Sub-theme: interrupted learning, eroding academic confidence, and deferral of research

Participants described a sequence rather than a single state. In their accounts, long interruptions to their learning came first. These interruptions eroded their confidence in their own academic ability. They then spoke of putting research tasks off, avoiding tasks they expected to find difficult, and losing further confidence each time a setback occurred.

*“During the pandemic, everything was conducted online, and I couldn’t concentrate at all. I had to sneak in theory classes while working, always worried that patients would notice. I had to figure out how to prepare for the proposal and midterm defense entirely on my own. After so much fragmented and interrupted learning, I started putting things off more and more. The longer I delayed, the less confident I felt” (P13).*

Some participants linked their permanent institutional posts to a reduced sense of urgency about academic work. They described research tasks as the ones that could always be postponed.

*“For us part-time master’s students in nursing, we have job security through our institutional posts. Even if we don’t finish the degree, it doesn’t affect our livelihood. It’s like being stuck in a state where you’re too tired to push forward but too uneasy to completely give up, so all these academic tasks just turn into electives that you postpone whenever you can” (P8).*

## 4 Discussion

This study set out to describe the challenges that part-time master’s students in nursing in China encounter in making research progress and the conditions in which those challenges arise. Our central reading of the accounts is that the delays participants described are not adequately explained by limited academic ability or insufficient motivation on the part of individual students. Across the sample, and at every stage of candidature, participants located their difficulties in conditions they did not control. These included the demands of three concurrent roles, the organization of clinical work, and the absence of any working link between hospital and university. They also included prevailing beliefs about the standing of nursing research and the timing of life events relative to program milestones.

These findings are consistent with those of Xu et al. (2025), whose participants described conflicting commitments, constraints of time and space, weakened interpersonal support, and perplexity about nursing research. The overlap is expected. Both studies drew on part-time postgraduate nursing students in China and used a descriptive design with reflexive thematic analysis. The present study differs in two respects that account for what it adds. First, Xu et al. (2025) excluded students who had not completed within the permitted period, whereas the present sample included two who had withdrawn. Their accounts, together with those of participants in the later years of candidature, made visible a sequence from early interruption through repeated deferral to disengagement. A sample of continuing students cannot show this sequence. Second, the present analysis located challenges at several ecological levels at once and examined how they were patterned by stage of progression. This drew attention to conditions that lie beyond the student’s immediate setting, in particular the absence of cross-institutional coordination and unequal access to research resources.

The three sub-themes of role conflicts and resource depletion correspond closely to the three forms of inter-role conflict (Greenhaus and Beutell, 1985). Fragmented time allocation is time-based conflict, in which time devoted to one role is unavailable to another. Mental exhaustion following frequent role switching is strain-based conflict, in which strain generated in one role impairs performance in another. The clash between clinical and academic value orientations is behavior-based conflict, in which the behaviors rewarded in one role are incompatible with those required in another. All three forms appeared, and participants at every stage described them. This is consistent with the finding of Goong et al. (2016) and Xu and Song (2016) that registered nurses pursuing advanced degrees experience work-family-school conflict as a compound rather than a single strain. *Role Theory* offers one frame for these accounts. In this framework, role overload arises when the demands or expectations attached to a set of roles exceed the resources, such as time and energy, that a person has available (Levinson et al., 1965). Participants, who straddled clinical practice and academic research, described these demands as acute. The role tensions they described also extended beyond the balance between work and family responsibilities. Participants managed overlapping responsibilities as clinical nurses, graduate students, and family members. They also contended with conflicting expectations from hospital administrators, academic supervisors, and broader sociocultural norms. The demands they described also included the emotional labor characteristic of nursing, such as caring for critically ill patients and reassuring anxious relatives (Feng et al., 2024) his labor adds to the load carried by the clinical role.

The findings suggest that slow research progress is not merely a matter of insufficient time. Participants associated it with cumulative mental strain and with constant switching between roles, which gradually drained their capacity to concentrate (Xu et al., 2025). The interruptions that characterize routine clinical work have been associated with higher mental workload among nurses (Shan et al., 2023). Participants described the frequent transitions between clinical, academic, and family roles in similar terms. Night-shift work has been linked to adverse health effects among nurses (Books et al., 2017). The mental workload of demanding clinical settings has been described as consuming nurses’ mental resources (Surendran et al., 2024) In these circumstances, improving time management alone is unlikely to be sufficient.

The structural barriers within the microsystem that participants described, particularly the systemic constraints of clinical practice settings, may reflect wider features of hospital staffing in China. Although the number of nurses in China has steadily increased, nurse staffing relative to physicians remains below the average reported for countries of the Organisation for Economic Co-operation and Development (OECD). By the end of 2024, China had 5.082 million practicing (assistant) physicians and 5.855 million registered nurses (National Health Commission of the People’s Republic of China, 2025). This is approximately 1.15 nurses per physician, against an OECD average of 2.5 (OECD, 2025). In large general hospitals in China, each nurse has been reported to be responsible for about 8 patients on a day shift and up to 23 on a night shift (Shen et al., 2020) Participants’ accounts of chronic understaffing are consistent with these figures.

This study also identified conditions associated with the limited effectiveness of supervisory interactions. Studies of doctoral supervision have described the supervisory relationship in terms of the supervisor’s influence and the interpersonal proximity between supervisor and student (Mainhard et al., 2009). The participants in this study described difficulties of a more structural kind. These included mismatches between supervisors’ academic backgrounds and students’ research directions, prolonged negotiation over research topics, and guidance that did not connect clinical experience with research. Zhang et al. (2024) describe supervision as operating in interpersonal, pedagogical, and academic registers. Participants described difficulties in all three. These data cannot establish whether such difficulties co-occur systematically. Delayed feedback and limited supervisory time have also been reported by postgraduate nursing students elsewhere (Havenga and Sengane, 2018). Supervisory support is among the most consistently identified influences on study progress in this population (Mudzi and Sepeng, 2023) Some participants described supervisors whose own field lay outside clinical nursing. This pattern merits further examination.

The weakening of peer support has a comparable structural source. Peer support among students in medicine and nursing typically consists of information exchange, mutual assistance, and emotional encouragement (Pereira et al., 2019). Peer and supervisor support are also among the factors identified as influencing postgraduate nursing students’ progress (Mudzi and Sepeng, 2023). The part-time students in this study were dispersed across institutions and shift patterns. The conditions under which peer support usually forms were therefore largely absent, and the function it usually performs was not available to them. Part-time doctoral students report a similar pattern. They describe weaker integration into the academic community and fewer opportunities for informal learning from peers than full-time students (Gardner and Gopaul, 2012). The present findings suggest that, for part-time students in a practice-based discipline, the isolation is compounded by shift work, which removes even the possibility of regular meeting times.

The absence of cross-institutional coordination at the mesosystem level was another distinctive feature of participants’ accounts. In Bronfenbrenner’s (1979) framework, the mesosystem consists of the links between the settings in which a person participates directly. For the participants in this study, the two principal settings, the hospital ward and the university, were separately governed. Participants described no arrangement, formal or informal, through which the demands of one were adjusted in light of the other. The distinctive feature of their mesosystem was therefore not a weak link but the absence of one. The consequences of that absence fell on the student. Students carried the costs of travel, of consecutive shifts followed by classes, and of reconciling two timetables. Curriculum design was a related but separate condition, located at the exosystem level. Theoretical coursework is concentrated in the first year of graduate study, which places a heavy academic load within a short time frame. Part-time students receive no corresponding reduction in clinical workload. Participants therefore described shouldering intensive clinical responsibilities alongside demanding academic tasks. Stakeholder accounts of master’s-level nursing training in China describe a framework built around full-time candidates (Shi et al., 2022). This is consistent with the mismatch participants described between the curriculum they were offered and the work they were doing. A configurational study of part-time master’s students in nursing in China similarly identified faculty-student interaction and a supportive work environment as conditions for engagement (Wang et al., 2024) he present accounts describe what their absence looks like from the students’ side.

Inequitable access to research resources has a similar institutional origin. In low and middle-income countries, including China, hospitals and universities frequently struggle to maintain database subscriptions in the face of high costs and budget constraints (Zhang et al., 2023). Participants described access to good materials and to expert advice at the proposal and writing stages as running through personal networks. For these students, a resource that a full-time program provides institutionally was contingent on whom they happened to know.

The crisis of disciplinary identity surrounding the value of nursing research was also a prominent feature of participants’ accounts. Participants described nursing research as marginal within a hospital culture dominated by biomedicine. Professional identity in nursing has been examined as a multidimensional construct shaped by professional context (Philippa et al., 2021) Participants described this marginalization as having become part of how they saw their own work, appearing as self-doubt and confusion about its value. We read these accounts as suggesting a self-reinforcing cycle. Doubts about the value of nursing research accompany limited engagement, and limited engagement leaves the peripheral status of nursing research unchallenged. Testing this reading would require longitudinal data.

Participants who described their studies as externally imposed also described letting research go once it became difficult. Their accounts suggest that motivation sustained mainly by external requirements offered little to draw on when the work became demanding. Zhang et al. (2022) report modest research motivation among postgraduate nursing students in traditional Chinese medicine colleges. Participants’ accounts here are consistent with that picture. A further observation deserves comment. Some participants who had enrolled at an employer’s instruction, and who described the structural conditions, nevertheless attributed their difficulties to themselves. The data support treating slow research progress as a trajectory shaped by conditions at several levels. Yet participants often explained it in the individual terms of interest, effort, or ability. That the two accounts diverge is itself a finding. It suggests that students may hold themselves responsible for delays whose sources lie largely outside their control. This pattern is consistent with the erosion of self-efficacy described in Muñoz’s (2021) concept analysis of graduate student self-efficacy. It is also consistent with Bandura’s (1997) account of how repeated failure experiences lower expectations of success. The sub-theme of utilitarian academic culture also bears on the concerns of the slow scholarship movement, although from the opposite direction. That movement defends deliberately unhurried inquiry as a response to productivity pressure (Hartman and Darab, 2012) The delays described here, by contrast, were involuntary. What the two share is a critique of academic environments organized around measurable output.

This study also identified converging demands during major life events. Most participants were at a life stage in which career advancement and family responsibilities overlapped. They described this overlap as sharpening role conflicts. *Life Course Theory* offers a frame for these accounts. It treats lives as unfolding through interdependent trajectories and transitions whose timing shapes later outcomes (Elder, 1998). It also draws attention to the consequences of a life transition coinciding with an institutional one. In this program, taught courses and foundational research training are concentrated in the first year. A participant whose pregnancy and childbirth fell in that year described missing training that was never made up. She traced later difficulties to that gap. A female participant described continuing to carry substantial caregiving responsibilities alongside her professional advancement. A scoping review of postgraduate nursing students’ study progress in other regions identifies family responsibilities among the influences on progress (Mudzi and Sepeng, 2023) It does not, however, foreground a gendered division of domestic labor. Women made up 12 of the 14 participants, in line with the composition of the nursing workforce in China. Gender therefore could not be examined as a variable in this study. These accounts describe a double obligation across the domestic and professional spheres. We do not present it as a general characteristic of women’s working lives in China.

Participants’ accounts also suggested a pattern we tentatively describe as perceived entrenchment of thinking after long clinical experience. We offer this as an interpretive proposition requiring further empirical examination. The pattern resembles what Dane (2010) terms cognitive entrenchment, a high level of stability in domain schemas that can accompany expertise and that varies with the task environment. Long clinical practice equips nurses with extensive experiential knowledge. Participants described it as also reinforcing habitual patterns of thinking. They regarded such routines as contributing to patient safety and operational consistency. They also described them as making new lines of research thinking harder to open. This reading points to a tension, within nursing as a practice-based discipline, between clinical standardization and academic innovation. It may offer a starting point for examining how workplace-based experiential learning shapes the capacity of part-time adult learners for scholarly inquiry. Because the pattern was constructed from participants’ self-descriptions, it would need to be examined with measures of cognitive flexibility before any stronger claim could be made.

The patterning of themes across stages of progression bears on how these conditions operate over time. The first four themes, from role conflict to macro-level cultural influences, were described at every stage. This suggests that the conditions they name are features of the part-time route itself rather than consequences of falling behind. The subtheme describing interrupted learning, deferral, and loss of confidence was elaborated mainly by participants in the later years and by those who had withdrawn. We read this as consistent with a cumulative account in which conditions present from the outset produce delay, and delay in turn produces the loss of confidence and disengagement that make further delay more likely. Because the stage comparison is cross-sectional and relies on retrospective accounts, it cannot establish that early-stage participants will follow the same course. It can only show that those who had fallen furthest behind described that course. The account is nonetheless consistent with research on attrition from graduate programs. In that research, departure is more often the endpoint of a gradual process of disengagement than a single decision (Lovitts, 2001). Part-time students are also at greater risk (Gardner and Gopaul, 2012).

The findings offer four propositions. First, conditions at the microsystem level feed role overload. Second, the absence of a mesosystem leaves the resulting conflicts with the student. Third, macrosystem beliefs shape how much priority research can claim and how students explain their own difficulties. Fourth, these conditions accumulate over the course of study.

## 5 Practical implications

### 5.1 Theoretical implications

This study bears on educational psychology in three ways. First, it suggests where the role conflicts of working adult learners in a practice-based discipline originate. *Role Theory* identifies conflict as arising when the expectations attached to concurrent roles are incompatible. The literature on working students has generally located the resulting strain in the individual’s allocation of time and energy across roles. Participants’ accounts located a substantial part of the conflict at the mesosystem level. They traced it to the absence of any institutional link between the hospital that set the clinical schedule and the university that set the academic one. In this reading, Role Theory names the strain and Ecological Systems Theory locates its source. The two frameworks are integrated in the proposition that inter-role conflict among parttime postgraduate students may be, in part, a product of institutional arrangements rather than of individual role management alone. Second, the findings indicate how several constructs central to the field operated in these accounts, and what each may imply for the design of support. Second, the findings indicate how several constructs central to the field operated in these accounts, and what each may imply for the design of support. Third, the proposition of perceived entrenchment of thinking after long clinical experience highlights a potential tension between workplace expertise and academic learning among adult learners. If the proposition holds, research training for experienced clinicians would need a pedagogy that treats their established schemas as a starting point to be examined rather than as an obstacle to be bypassed. The proposition was constructed from participants’ own accounts of how they approach problems, not from any measure of cognitive flexibility. It is therefore offered for further conceptual and empirical attention.

### 5.2 Practical implications

At the level of the clinical workplace, hospitals and nursing departments could designate protected research time within the roster for nurses enrolled in postgraduate study, as is done for clinician-researchers in medicine (Lingard et al., 2017). They could also treat enrollment as a matter for workload planning rather than for the individual nurse to absorb, and reduce interruptions within protected time. These measures respond to the theme of role conflicts and resource depletion, in particular fragmented time and mental exhaustion, and to the theme of structural barriers within the microsystem.

At the level of the relationship between hospital and university, the theme of insufficient support from mesosystem and exosystem levels points to a joint mechanism established by the two institutions. The mechanism could schedule coursework around clinical rosters, share information about students’ progress, and give part-time students access to the university’s literature databases irrespective of their hospital’s subscriptions. Joint appointments and formal partnerships between hospitals and universities have been used in nursing to align clinical and academic expectations (Beal, 2012) and offer one model for the coordination that participants described as absent.

At the level of the program, universities offering master’s programs in nursing could develop a part-time route with its own curriculum rather than a full-time curriculum delivered on a part-time timetable. The route could offer structured first-year research training built on clinical problems, supervision matched to students’ clinical research interests, formal induction for students entering from full-time employment, and peer groups scheduled around shifts. These measures respond to the theme of insufficient support from mesosystem and exosystem levels, and to the theme of structural barriers within the microsystem, including the loss of peer support that dispersal causes.

At the level of professional and institutional culture, the bodies that set criteria for nurses’ professional title advancement and for hospital research assessment could recognize nursing research on its own terms. They could weight the quality and clinical relevance of research over its quantity. Such recognition would address the doubts about the standing of nursing research described under the theme of macro-level cultural influences, and would remove one of the conditions under which enrollment is undertaken for credentials alone.

## 6 Limitations

Several limitations should be acknowledged. First, participants were drawn from a single medical university, and therefore from a single training program. The institutional conditions described are features of that program, including the concentration of coursework in the first year, the six-year limit on candidature, and the absence of any reduction in clinical workload. Other programs may structure the part-time route differently, so the findings should be read against the contextual detail provided rather than as a description of part-time nursing programs in China as a whole. Second, the design relied primarily on a single interview with each participant, conducted between October and December 2025. Contact with the data and participants continued until February 2026, but the study could not follow how the challenges evolve over the course of the program. The cross-stage matrix is therefore cross-sectional, rests on retrospective accounts, and cannot substitute for longitudinal observation. Third, several team members were known to participants as colleagues, and interviews took place at participants’ workplaces. This may have encouraged participants to frame their difficulties in terms they expected the team to accept. The reflexive procedures described were intended to reduce this influence, although they cannot eliminate it. Fourth, the first interviews were relatively short (27–39 min), which may have limited the depth of some accounts. Three supplementary interviews addressed gaps identified in the preliminary analysis. Constructs such as academic self-efficacy and entrenchment of thinking were nonetheless inferred from self-report rather than measured.

Fifth, the sample included only two men and two students who had withdrawn. Experiences specific to these groups therefore rest on few accounts. Snowball sampling was used specifically to reach former students, and one of them took part in member checking. Sixth, the extracts were translated from Mandarin Chinese into English, and some nuance of participants’ wording may not have carried across. Translation followed the procedures adopted for this study, which reduce but cannot remove this risk. Seventh, because self-reported slow research progress was an inclusion criterion, the study did not include part-time students who were progressing as expected. It therefore cannot identify which of the conditions described distinguish students who fall behind from those who do not. Eighth, only students were interviewed. Supervisors, hospital managers, and program administrators, whose decisions shape several of the conditions described, were not consulted. Their accounts of the same conditions may differ.

## 7 Conclusion

This study asked how slow research progress among part-time master’s students in nursing in China should be understood. Participants’ accounts support treating it as a trajectory rather than as an individual trait or behavior. In these accounts, it took the form of a sustained delay in reaching thesis milestones. This delay was shaped by conditions that operated simultaneously at several levels. They lay in the clinical workplace, the relationship between hospital and university, the design of the program, beliefs about the standing of nursing research, and the timing of life events relative to program requirements. Students at every stage of candidature described these conditions. Their accumulation over time, from early interruption through repeated deferral to loss of confidence, was most fully described by those who had fallen furthest behind. No single factor accounted for the delays. Participants sometimes explained their own difficulties in individual terms, but these explanations sat alongside accounts that located the sources of those difficulties outside their control. The findings describe what the part-time route looks like from the student’s side in one program. They indicate that support for part-time postgraduate learners in nursing will need to act on institutional arrangements, and not only on students, if research progress is to improve.

## Data Availability

The original contributions presented in the study are included in the article/supplementary material, further inquiries can be directed to the corresponding author.
